# The Shadows of Normal Flora on Minor Wounds: A Case Report of an Uncommon Pathogen With Potentially Lethal Consequences

**DOI:** 10.7759/cureus.59648

**Published:** 2024-05-04

**Authors:** Khurram Arshad, Farman Ali, Yazan Alamro, Rabia Latif, Antoine Egbe Bessong Tabot

**Affiliations:** 1 Internal Medicine, Corewell Health East Dearborn, Dearborn, USA; 2 Medicine, St. John Hospital and Medical Center, Detroit, USA; 3 Internal Medicine, Beumont Hospital Dearborn, Dearborn, USA; 4 Internal Medicine, McLaren Hospital, Flint, USA

**Keywords:** case report, mortality rate, intravenous antibiotics, antimicrobial resistance, infective endocarditis, staphylococcus lugdunensis

## Abstract

Staphylococcus lugdunensis is a gram-positive, coagulase-negative organism, typically found in the normal skin flora, predominantly colonizing the perineal region. It has gained recognition as an opportunistic pathogen capable of causing severe infections.

This manuscript presents a case study of a 75-year-old female with multiple comorbidities, including hypertension, hyperlipidemia, atrial fibrillation on Xarelto, type 2 diabetes mellitus, hypothyroidism, and a bioprosthetic aortic valve. The patient exhibited symptoms of fever, chills, and lethargy following a dog scratch that resulted in wounds on the left lower extremity. Despite initial negative findings in the drug screen and unremarkable workup for other infectious etiologies, the patient's clinical course revealed the presence of S. lugdunensis in the blood cultures. Timely intervention with broad-spectrum intravenous antibiotics and a six-week course of cefazolin led to significant improvement without recurrence.

Staphylococcus lugdunensis, previously considered a relatively benign microorganism, has become a significant player in infectious diseases, particularly causing skin and soft tissue infections and infective endocarditis (IE). It is considered an aggressive pathogen, especially in chronic immunocompromised personnel, with a high potential for morbidity and mortality. S. lugdunensis was found to be the fourth most common cause of IE. The manuscript discusses the epidemiology, clinical presentation, and management of S. lugdunensis infections, emphasizing the importance of early recognition and treatment to prevent potentially fatal outcomes.

## Introduction

Staphylococcus lugdunensis, identified as a gram-positive, coagulase-negative organism [1], typically found in the normal skin flora, predominantly colonizing the perineal region [2,3], has gained recognition as an opportunistic pathogen capable of causing severe infections. While part of the normal skin flora, particularly in the perineal region, its ability to lead to life-threatening conditions such as infective endocarditis (IE) highlights the need for increased awareness and understanding of this microorganism [4]. This case aims to provide an overview of S. lugdunensis, its prevalence, and the potential clinical implications associated with its infections.

## Case presentation

**Figure 1:**
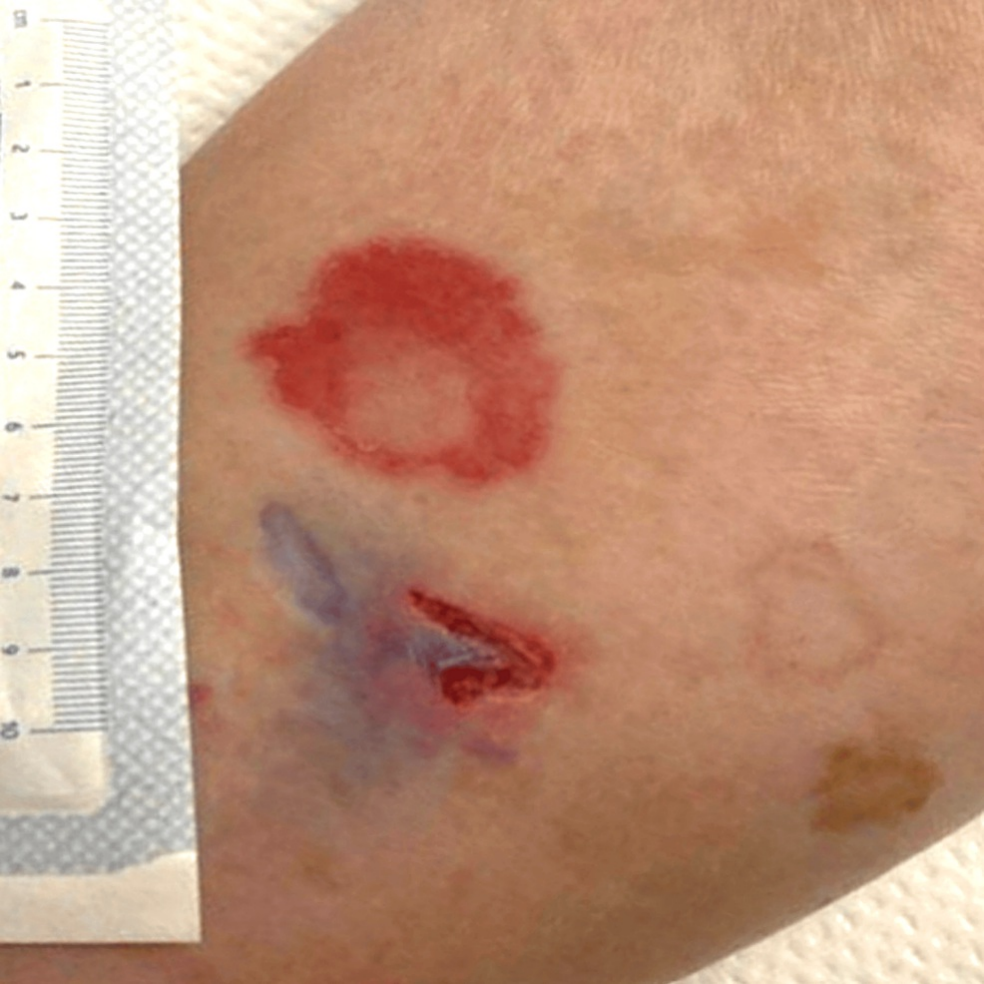
Two acute healing scratch wounds measure approximately 1 and 3 cm with no evidence of exudates or soft tissue infection.

A 75-year-old female with a medical history encompassing hypertension, hyperlipidemia, atrial fibrillation managed with Xarelto, type 2 diabetes mellitus, hypothyroidism, and a bioprosthetic aortic valve presented with symptoms of fever, chills, and lethargy. The onset of these symptoms followed a dog scratch to her left lower extremity two weeks prior, resulting in wounds near the shin region (Figure 1). Despite a negative drug screen and an exhaustive workup for other potential infectious causes, the patient's blood cultures unexpectedly revealed the presence of Staphylococcus lugdunensis.

**Figure 2:**
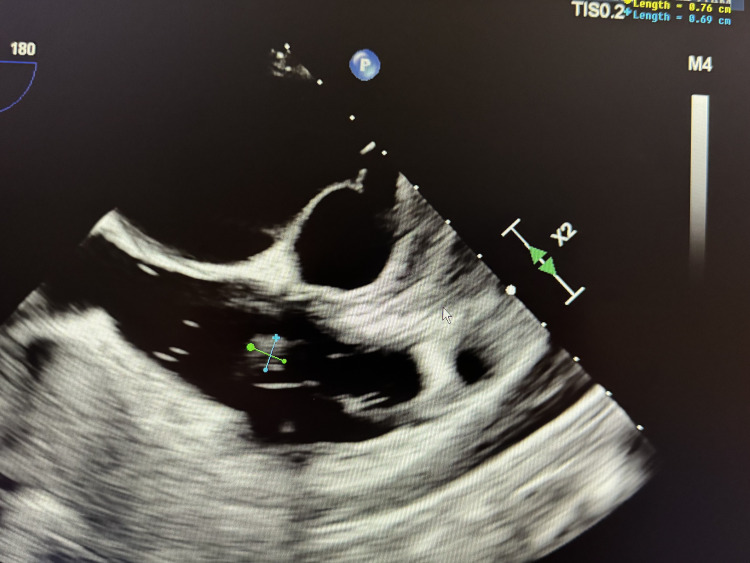
Transesophageal echocardiogram; the marked area shows vegetation in the tricuspid area that is pathognomonic for infective endocarditis.

The interdisciplinary care team closely monitored the patient throughout her hospitalization, including cardiology, infectious disease, and endocrinology. A transesophageal echocardiogram (TEE) revealed a mobile density attached to the tricuspid valve, measuring 0.4 cm, indicative of vegetation (Figure 2) without any abcess or tricupsid regurgitation. Subsequent blood cultures returned negative, and the patient exhibited improvement without any recurrence of symptoms. Following a thorough assessment, she was discharged with a prescription for intravenous antibiotics, to be administered over a six-week period. This comprehensive approach to management proved effective in the patient's recovery. After six weeks of the antibiotic course patient was followed by repeat TEE which came out unremarkable and showed resolution of vegetation.

## Discussion

Staphylococcus lugdunensis, previously considered a relatively benign microorganism, has become a significant player in infectious diseases, particularly causing skin and soft tissue infections and infective endocarditis [4]. It is considered an aggressive pathogen, especially in chronic immunocompromised personnel, with a high potential for morbidity and mortality. S. lugdunensis was found to be the fourth most common cause of IE [5]. The infection can occur in native and prosthetic valves and can be associated with large vegetations, with a higher prevalence for mitral valves than other valves [6]. This case delves into the epidemiology of S. lugdunensis, its clinical presentation, and the challenges associated with its diagnosis and management.

The presented case of a 75-year-old female with multiple comorbidities illustrates the diverse clinical manifestations of S. lugdunensis infections. The patient's history of a dog scratch leading to wounds on the left lower extremity is noteworthy, as it emphasizes the potential for this pathogen to cause severe infections even through seemingly minor skin breaches [4]. While most of the reported cases have been reported in patients with vascular access, in our case, the infection resulted from a minor wound.

In the presented case, Staphylococcus lugdunensis was identified in blood cultures, prompting immediate intervention with broad-spectrum intravenous antibiotics and a six-week course of cefazolin. This favorable outcome highlights the critical significance of early detection and targeted antimicrobial therapy in effectively managing S. lugdunensis infections. Despite its susceptibility to a wide range of antibiotics compared to other coagulase-negative staphylococci, S. lugdunensis is resistant to penicillin due to β-lactamase production. Although the emergence of methicillin resistance is rare in the USA, documented cases emphasize the need for vigilance. Clindamycin and erythromycin serve as viable alternative treatments, boasting low resistance prevalence in cases of S. lugdunensis infection [7,8].

The discussion also highlights the potential complications associated with S. lugdunensis infections, including fatal myocarditis, osteomyelitis, and central nervous system infections leading to cerebrovascular accidents [9,10]. Valvular pathology involving S. lugdunensis typically results in bulky leaflet vegetations, predominantly affecting left-sided heart valves [4]. A systematic review revealed that 94.7% of all IE cases due to this organism were left-sided, with the mitral valve being most commonly affected (52.5%), followed by the aortic valve (37%) [9].

Despite its susceptibility to a broad range of antibiotics compared to other coagulase-negative staphylococci, S. lugdunensis exhibits resistance to penicillin due to β-lactamase production. The chosen antibiotic for the presented case was intravenous nafcillin. Although methicillin resistance is rare in the USA, documented cases highlight the importance of alternative treatment options such as clindamycin and erythromycin, which exhibit low resistance prevalence [7,8]. A prospective cohort study underscores the gravity of S. lugdunensis IE, revealing mortality rates of up to 42% in native valves and 78% in prosthetic valves. Early surgical intervention, along with an aggressive intravenous antibiotic regimen, is recommended to reduce mortality in these patients significantly [4].

## Conclusions

The presented case underscores the importance of recognizing Staphylococcus lugdunensis as a potential pathogen capable of causing severe infections, especially in individuals with predisposing comorbidities. As demonstrated in our case, early diagnosis and intervention are crucial for successful management and prevention of adverse outcomes. This manuscript raises awareness among healthcare professionals about the clinical significance of S. lugdunensis and advocates for vigilance in its detection and appropriate therapeutic strategies.
